# Fabrication, spectroscopic inspection, biological evaluation of some transition metal complexes with *bis*- azomethine ligand and γ-ray irradiation dose effect

**DOI:** 10.1038/s41598-025-08660-5

**Published:** 2025-07-16

**Authors:** Hanaa A. El-Boraey, Ohyla A. EL-Gammal

**Affiliations:** 1 Department of Chemistry, Faculty of Science, Menoufia University, Shebin El- Kom, 32511 Egypt; 2 Department of Pathology, University Hospital, Menoufia University, Shebin El- Kom, 32511 Egypt

**Keywords:** Metal complexes, Spectroscopic characterization, X-ray diffraction, Anticancer, Microbial activity, Gamma irradiation, Chemical biology, Chemistry, Materials science

## Abstract

A new *bis-* azomethine ligand N′,N′′′-(2,2′-(((1E,1′E)-cyclohexa-2,5-diene-1,4-diyl*bis*(methanylylidene))*bis*(azanylylidene))*bis*(benzoyl))di(picolinohydrazide) and its Cd^+2^ (**1**), Hg^+2^ (**2**) and Co^+2^ (**3**) complexes have been fabricated. Via FT-IR, UV/Vis., ^1^H-NMR spectroscopy and XRD, in addition to magnetic moment, elemental, molar conductance and thermal analysis, the structure of synthesized compounds was explored. The parent complexes (**1,2**) have been irradiated by ^60^Co radioactive source γ-rays at room temperature with dose values of 150 and 200 kGy to get irradiated samples (**1R** and 1R*) and (**2R** and 2R*), respectively. The structural of the irradiated samples were studied by the same techniques as for the parent complexes. The cytotoxicity of the ligand and its Cd^+2^ and Hg^+2^ complexes against breast (MCF-7) and hepatocellular cancerous cells (HepG-2) was evaluated. The chelates are significantly more potent compared to the ligand. Moreover, these compounds have been screened for their antimicrobial potency. The finding showed that the parent metal complexes demonstrated promising antimicrobial activity against gram −ve (*Escherichia coli*) and gram + ve (*Staphylococcus aureus*) bacterial species and two fungal species (*Candida albicans* and *Aspergillus nigar*) as well. The complexes exhibited greater antimicrobial potency than Ampicillin and Nystatin.

**Supplementary Information:**

The online version contains supplementary material available at 10.1038/s41598-025-08660-5.

## Introduction

Nowadays, the ionizing radiation usage gradually increasing with the development of technology. Many nuclear, medical and industrial fields employ radiations like gamma and X-rays^1^. Also, ionizing radiation can be used for exploring and advanced purposes, in addition to radiation sterilization of biomolecular materials^2^. Gamma irradiation can change the biological characteristics and structure of organic compounds^3,4^. The morphology and physio-chemical characteristics of the materials may be significantly impacted by high energetic radiations, which might also alter the materials’ sensing, optical, structural and electrical properties^5^. Gamma rays lead to enhancement of the stability of some irradiated compounds compared to the nonirradiated ones. Further, the PXRD patterns cleared that the crystallinity of nanoscale particles was increased after gamma irradiation^6^. The potential impacts of ionizing radiation on the composition structure were not studied by the scientists, who were only attracted to the radiation shielding properties^7^. γ-Rays are applied on inorganic salts, organic polymers and metal complexes due to their impact on the chemical properties such as chemical structure and biological activity^8,9^. Accordingly, several studies have been reported to explore the impacts of gamma radiation on the properties of these materials at a wider range of doses^10^.

The Schiff base ligands are a vital class of biologically active drug molecules due to their wide range of pharmacological fields^11^. They able to combine with versatile transition metal ions to produce stable complexes. Transition metals are of great importance in several application areas and showed a broad range of applications in many areas such as industrial, pharmacological’, including anticancer, antifungal, antioxidant as well as antibacterial agents and in numerous fields of chemistry and agriculture^12–18^. When complexation of organic molecules with metal ions occurs, the biological and therapeutic properties of both ligand moiety and the metal are significantly altered^19,20^. Schiff base complexes display higher biological activity compared to free ligands; also, they serve as critical intermediates in the synthesis of natural products^21^. Recently, the interest in the interaction between DNA and transition metal complexes that include multidentate aromatic ligands, primarily N-containing ligands had been increased. This is due to their potential used in therapeutic treatments, making them emerging probes of DNA configuration and structure^20^. Also, hydrazine, hydrazone, and their derivatives are potential group of compounds due to their significant biological and pharmacological activities as antitumor, antifungal, anti-inflammatory, antibacterial, DNA binding and antimalarial agents. The major concerns of developing metal chelate as a potential drug are their potential metabolic as well as toxicity problems. The accumulation of heavy metal ions in the human body can cause health problems such as hypertension, brain damage, kidney failure and cancer. The physicochemical properties and pharmacological metabolic profile of these of metal-based drugs expected to safe by tuning their metabolism with chemoprotective agents^22–24^. These kinds of chelates interact with DNA mainly through non-covalent modes, namely, intercalation, major and minor grooves, and electrostatic binding. It has been demonstrated that non-covalently bound anticancer drugs have fewer adverse effects than covalently bound anticancer drugs. In response to the growing need for new therapeutic agents in this study, a new Schiff base ligand: N′,N′′′-(2,2′-(((1E,1′E)-cyclohexa-2,5-diene-1,4 diylbis(methanylylidene))*bis*(azanylylidene))bis(benzoyl))di(picolinohydrazide) (L) was synthesized by condensation of 2-aminobenzoyl)isonicotinohydrazide with terephthaldehyde. The ligand was allowed to react with Cd^+2^, Hg^+2^ and Co^+2^ ions to give the corresponding parent complexes. The synthesized ligand and its complexes were evaluated using numerous spectral methods, like FT-IR, ^1^H-NMR, UV/Vis., elemental analysis, magnetic, thermal properties and powder X-ray diffraction. Furthermore, the current work highlights the effect of two distinct γ-irradiation dosages (150 and 200 kGy) on the physicochemical properties of Cd^+2^, Hg^+2^ complexes. In addition, the antitumor property of the ligand and its Cd^+2^, Hg^+2^ complexes were evaluated in vitro against different cell lines, namely the hepatocellular carcinoma (HepG-2) and breast cancer (MCF-7) cell line. Also, the antimicrobial property of the synthesized ligand and its selective metal complexes towards *Staphylococcus aureus* and *Escherichia coli* bacteria and *Candida albicans* and *Aspergillus nigar* fungi were evaluated.

## Experimental

### Analysis and physical measurements

All instruments-generated spectra for all the analyses are provided in the supplementary materials^25–29^.

### Chemicals

All chemicals utilized were obtained from Sigma-Aldrich. CdBr_2,_ HgCl_2_ and Co(NO_3_)_2_.6H_2_O were purchased from E. Merck, Nasr City, Egypt. All reagents were used as it is. Analyses of the metal ion and chloride contents were performed by per standard procedures^30^.

### Procedure for the preparation of the ligand

The ligand was synthesized by refluxing 1:2 molar ratio ethanolic solution of (2-aminobenzoyl)isonicotinohydrazide (2.78 mmol, 1 g) with terephthaldehyde (1 mmol, 0.25 g) in ethanol (20 mmol) under constant stirring for a period of 5–6 h, until a precipitate formed (**L**). The isolated yellowish- green product was then filtered off, washed numerous times with ethanol and desiccated in vacuo.

Color: Yellowish- green; Yield: 80%; Anal. found for C_34_H_24_N_8_O_4_ (%): C, 61.29; H, 4.50; N, 16.93. Calcd. (%): C, 61.63; H, 4.53; N, 16.92. FT-IR (KBr, cm^−1^): 1698(s),1642 ʋ(C = O), 1613 ʋ(–HC = N–), 3204–3184 υ(N–H), 3425 υ(-OH/H_2_O). UV/Vis (λ_max_, nm): 374, 216.

### Synthesis of the metal complexes

(0.5 g, 1.39 mmol) of the ligand (**L**) in 20 mL ethanol was added gradually to ethanolic solution of CdBr_2_ (0.5 g, 1.8 mmol) (**1**), HgCl_2_ (0.5 g, 1.8 mmol) (**2**) or Co(NO_3_)_2_.6H_2_O (0.5 g, 2.1 mmol) (**3**). The reaction was performed in stoichiometric ratio of 2 metal: 1 ligand and refluxed with constant stirring. The products obtained were separated out by filtration and dried in a vacuum desiccator.

- [CdL(OH)_2_(H_2_O)_2_] complex (**1**)

Color: Yellow; Yield: 80%; D.T.: 250 °C. Conductivity (ohm^−1^ cm^2^ mol^−1^) in DMF: 34.

Anal. found for C_34_H_30_N_8_O_8_Cd (%): C, 52.17; H, 4.04; N, 14.83; M, 12.55. Calcd. (%): C, 51.65; H, 3.79; N, 14.18; M, 12.43. FT-IR (KBr, cm^−1^): 1705, 1663 ʋ(C=O), 1609 ʋ(–HC = N–), 3354,3286 υ(N–H), 1491 [ν(C–N)] + δ(N–H)], 595 ʋ(M–N), 448 ʋ(M–O), 3445 ʋ (-OH/H_2_O). UV/Vis (λ_max_, nm): 510,380,220. µ_eff_ (B.M.), diamagnetic.

- [HgLCl(H_2_O)_3_]Cl complex (**2**)

Color: Yellow; Yield: 85%; D.T.: 250 °C. Conductivity (ohm^−1^ cm^2^ mol^−1^) in DMF: 77.2.

Anal. found for C_34_H_30_N_8_O_7_HgCl_2_ (%): C, 43.33; H, 3.17; N, 11.97; M, 13.40; Cl, 15.00. Calcd. (%): C, 43.70; H, 3.21; N, 11.99; M, 13.76, C l,15.38. FT-IR (KBr, cm^−1^): 1700, 1660 ʋ(C = O), 1610 ʋ(–HC = N–), 3287,3191*υ*(N–H), 1492 [ν(C–N)] + δ(N–H)], 597 ʋ(M–N), 451 ʋ(M–O), 3439 ʋ (-OH/H_2_O). UV/Vis (λ_max_, nm): 515,300,210. µ_eff_ (B.M.), diamagnetic.

- [Co_2_L(NO_3_)_4_(H_2_O)_4_].4H_2_O complex (**3**)

Color: Light- brown; Yield: 73%; D.T.: 164 °C. Conductivity (ohm^−1^ cm^2^ mol^−1^) in DMF: 34.5.

Anal. found for C_34_H_40_N_12_O_12_Co_2_ (%): C, 36.63; H, 3.72; N, 12.64; M, 17.68. Calcd. (%): C, 36.50; H, 3.58.; N, 12.52; M, 17.33. FT-IR (KBr, cm^−1^): 1678,1638 ʋ(C = O), 1614 ʋ(–HC = N–), 3313,3204 υ(N–H), 1489 [ν(C–N)] + δ(N–H)], 585 ʋ(M–N), 500 ʋ(M–O), 3382 ʋ (–OH/H_2_O). UV/Vis (λ_max_, nm): 600,510,374,318,212. µ_eff_ (B.M.), 3.28/Co^2+^.

### Gamma-ray irradiation

Samples of Cd^+2^, Hg^+2^ complexes (**1,2**) were exposed to γ-ray at two different doses (150 and 200 kGy) by a ^60^Co source, at a dose rate of about 2.8 kGy h^-1^ at room temperature, cell type GE-4000 A at the Atomic Energy Authority of Egypt, Nasr City (designated as **1R, 2R** for 150 kGy dose and as 1R*, 2R* for 200 kGy dose values, respectively**)**. Following their removal from the radiation field, infrared, electronic spectra, molar conductivities, magnetic susceptibilities, PXRD and TG/DTG for the γ-irradiated samples were investigated by the same procedures applied for the parent complexes^31,32^.

### Pharmacology

#### In vitro cytotoxicity evaluation

In order to evaluate the cytotoxicity potency of the synthesized ligand and its Cd^+2^ and Hg^+2^ complexes, viability assay was applied using breast (MCF-7) and liver (HepG-2) cancer cell lines and comparing the IC_50_ value with cisplatin as standard. The cell lines were obtained from the American Type Culture Collection (ATCC, Rockville, MD) and investigated at the Regional Center for Mycology and Biotechnology, Al-Azhar University, Cairo, Egypt. Procedure of antitumor assay is given in the supplementary materials^33,34^.

#### Antimicrobial assay

By using agar well diffusion process, the in vitro antimicrobial property of the investigated compounds was evaluated^35^. *Staphylococcus aureus* (Gram + ve bacteria), *Escherichia coli* (Gram -ve bacteria) was chosen to study the antibacterial activity utilizing nutrient agar medium. The antifungal property of the investigated compounds was screened towards *Candida albicans and Aspergillus niger* utilizing Sabouraud dextrose agar medium. The antimicrobial activities of the investigated compounds were compared with standard references Ampicillin and Gentamicin (antibacterial) and Nystatin (antifungal). DMSO was used to prepare the negative control. The concentration of the compounds was 15 mg/mL against microbial strains. Method of testing is showed in the supplementary materials.

##### Statistical analysis

Differences between samples in the same type of bacteria (or fungi) were analyzed using one way analysis of variance (ANOVA), followed by Duncan multiple comparisons test using SPSS package version “22” for Windows. Values are represented as mean ± S.E. and *p* < 0.05 was considered statistically significant, *p* < 0.01 was considered highly significant and *p* < 0.001 was considered very highly significant.

## Analysis and discussions

### Physicochemical data of the prepared compounds

The reaction of ((2-aminobenzoyl) isonicotinohydrazide) with terephthaldehyde in ethanol leads to the present ligand: N′,N′′′-(2,2′-(((1E,1′E)-cyclohexa-2,5-diene-1,4-diyl*bis*(methanylylidene))*bis*(azanylylidene))*bis*(benzoyl))di(picolinohydrazide) (**L**), the ligand was allowed to interact with CdBr_2_, HgCl_2_ or Co(NO_3_)_2_.6H_2_O salts in 2 metal:1 ligand molar ratio to yield mononuclear Cd^+2^, Hg^+2^ complexes (**1,2**) and binuclear Co^+2^ complex (**3**). Full characterization of the produced compounds was investigated by applying spectroscopic and analytical techniques. All compounds are air stable at lab temperature (25 ± 2 °C). The ligand is soluble in ethanol whereas the complexes are reactive in DMSO and DMF and inert in other solvents. The molar conductivity measurements were performed with 1 × 10^–3^ M DMF at 37 °C, the recorded values are 34, 34.5 Ω^−1^ cm^2^ mol^-1^ for complexes (**1,3**), respectively, suggesting non-electrolytic behavior of these complexes, whereas for complex (**2**) it is 77.2 Ω^−1^ cm^2^ mol^−1^, conforming clearly its ionic nature^36^.

### Color and molar conductance of irradiated samples

The complexes’ color is not altered upon γ-irradiation. No color difference was detected, which might be explained by the surface and chemical stability of these metal complexes, that remain unchanged even after being exposed to γ-ray irradiation. The *Λ*_*m*_ values of the metal complexes subjected to γ-irradiation displayed slight change and still acquire non-electrolytic behavior (*Λ*_*m*_ = 40,40 Ω^−1^ cm^2^ mol^−1^) for samples 1R, 1R*, respectively. Samples 2R,2R* still behave as electrolytes (*Λ*_*m*_ = 73.5,70 Ω^−1^ cm^2^ mol^−1^)^37^.

### Spectroscopy

#### ^1^H-NMR spectra of ligand and Hg^+2^ complex

**Fig. 1:**
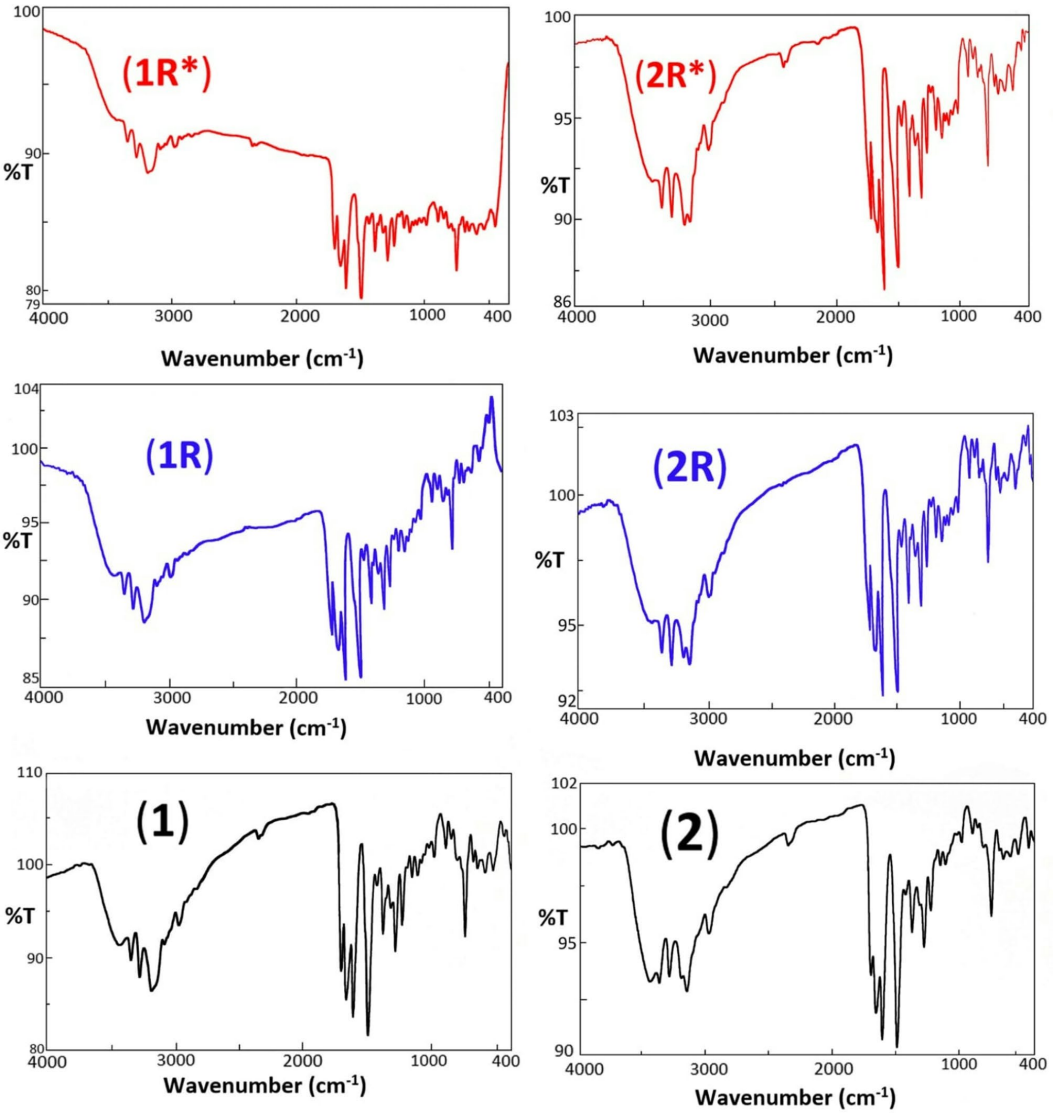
FT-IR spectra of complexes (1,1R,1R*) and (2, 2R,2R*).

**Fig. 2:**
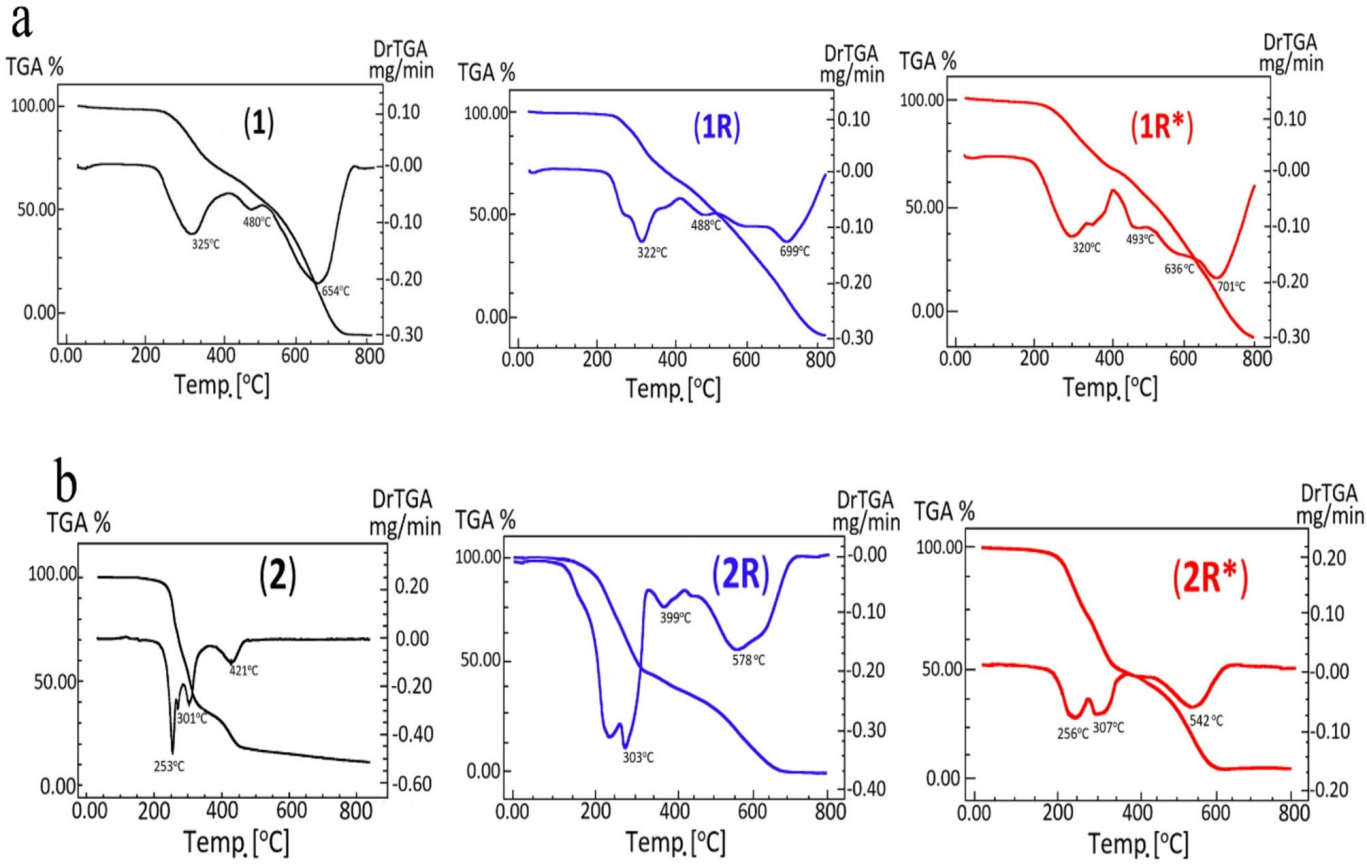
TG/DTG curves of complexes **a:(1,1R** and 1R*) and b: (2,2R and 2R*).

The ^1^H NMR spectra were carried out for the ligand, Hg^+2^ complex (**2**) as well as its γ-irradiated samples (2R,2R*) in DMSO-*d*_*6*_ medium (Figs. 1S, 2S). The free ligand has ^1^H NMR signals for amide (–CO–NH–) protons at δ 11.084 ppm, azomethine protons (–CH = N–) at δ = 8.890 ppm, pyridine and aromatic ring protons at δ = 6.220–8.115 ppm, after the complexation with Hg^+2^, the azomethine (–CH = N–) changed to downfield at δ = 8.774 ppm, the signal due to amide protons together with pyridine and aromatic ring protons has not registered any appreciable change compared to that of the free ligand^3,38^. The finding indicated that only the azomethine(–CH = N–) nitrogen are participating in coordination. Signals observed at 4.032–4.140 ppm in the proton NMR spectra of the ligand and complex (**2**), assignable to water molecules.

The ^1^H-NMR spectra of the γ-irradiated Hg^+2^ samples at dose value of 150 kGy (**2R)** and 200 kGy (2R*) (Fig. 2S), exhibited signals due to azomethine protons at 8.808 ppm in complex (**2R**) and at 8.813 ppm in complex (2R*). Also, the amide protons, the pyridine and aromatic ring protons have not registered any appreciable change compared to that of parent Hg^+2^ complex (**2**). The chemical shift as well as intensity showed slight change due to the effect of the applied γ-ray doses.

#### Infrared spectral studies

The bands in the FT-IR spectrum of the free ligand appeared at (1698, 1642), 1508 cm^−1^ related to ν(C = O) and [ν(C–O) + δ(N–H)], at 1284 cm^−1^ amide III; and at 752 cm^−1^ φ(C = O)^39,40^. The ligand FT-IR spectrum also showed strong splitted band in the 3204–3185 cm^−1^ range due to the stretching frequency of the secondary amide nitrogen ν(NH)^8,38^. The band at 1613 cm^−1^ is assignable to the ν(CH = N) stretching vibrations that confirmed the Schiff base formation^41^. The FT-IR spectrum also showed splitted peak at 3425 and at 2080–1996 cm^−1^ range, owing to ν(O–H) and intra- and/or intermolecular hydrogen bonds^42,43^.

In the FT-IR spectra of Cd^+2^, Hg^+2^ complexes (**1,2**), the ν(C = O) and ν(NH) registered higher shifted from their initial location compared to the free ligand, repealing the complexation via amide oxygen or amide nitrogen. The ν(CH = N) absorption band undergoes lower shift compared to the Schiff base ligand indicating coordination with the metal ion through azomethine nitrogen atoms. For Co^+2^ complex (**3**), two strong ν(C = O) peaks are observed at 1678 and 1638 cm^−1^. The observed lower shift of the first band from1698 to 1678 cm^−1^ together with the shift of ν(NH) bands indicated the coordination of the amide nitrogen (N^1^, N^1’^), while the second ν(C = O) band was observed approximately at the same frequency as for the metal free ligand. Definite evidence of the coordination is also revealed by the presence of new bands in all spectra of the complexes appeared at the range of 601–585 cm^−1^ that corresponded to ν(M − N) stretching vibrations^44,45^. The vibration peak occurred at 3425 cm^−1^ in the spectrum of the free ligand and at 3445–3354 cm^−1^ in the complexes is owing to OH/H_2_O stretching. Moreover, coordinated H_2_O molecules and/or hydroxyl group showed new medium bands of *ρ*r(H_2_O) rocking vibration at the region of 841–781 cm^−1^ and of *ρ*w(H_2_O) wagging vibration at 690–650 cm^−1^^38,43^. This coordination mode is further confirmed by the presence of new bands in the range of 500–448 cm^−1^ due to ν(M–O) vibrations in all chelates^46,47^. Co^+2^ complex (**3**) showed additional bands at 1421, 1305, 1040 cm^−1^, assignable to coordinated nitrate ion^48^. Therefore, the above data showed that the ligand coordinated in a neutral bidentate manner through the two azomethine nitrogen atom in the complexes (**1,2**) or neutral quadridentate one through (N^1^), (N^1’^) and two azomethine nitrogen atom in the binuclear complex (**3**).

### Infrared spectra of γ-irradiated samples

The FT-IR spectra of γ-irradiated samples (1R, 1R*) and (2R, 2R*) are compared with their parent complexes (**1**,**2**) (Fig. 1). The FT-IR for irradiated samples showed slight change in the band frequency as well as band intensity of some significant peaks, especially that of ν(OH/H_2_O): (3435, 3354 cm^−1^) for (1R, 1R*); (3362, 3363 cm^−1^) for (2R, 2R*); ν(C = O): (1703, 1656; 1703, 1654 cm^−1^) for (1R, 1R*), (1700, 1656; 1700, 1650 cm^−1^) for (2R, 2R*); [ν(C–O) + δ(N–H)], (1490; 1490 cm^−1^) for (1R, 1R*), (1490, 1491 cm^−1^) for (2R, 2R*); ν(M − O), (458; 452 cm^−1^) for (1R, 1R*), (456; 452 cm^−1^) for (2R, 2R*) and ν(M − N), (596; 594 cm^−1^) for (1R, 1R*), (598; 601 cm^−1^) for (2R, 2R*). The observed shift is probably due to general deformity of the lattice planes by applied gamma doses^49^.

#### Electronic spectroscopy and magnetic susceptibility measurements

The electronic spectra of the ligand and its complexes (**1–3**) were checked out in DMF as a solvent at the wavelength region 200–1100 nm. The absorption spectrum of the ligand showed the first high energy band at 374 nm, that is attributed to n → π* transitions of azomethine and carbonyl chromophores. The peak observed at 216 nm is reasonably accounted for π → π* transition of the aromatic and pyridine rings, these absorption bands suffered shift after complexation.

The UV/Vis. spectra of Cd^+2^, Hg^+2^ complexes showed bands at 510, 380 and 220–210 nm, assignable to charge transfer (MLCT), n → π* and π → π* transitions, respectively. No bands for d–d transitions were observed in these complexes consistent with the (d^10^) configuration. As predicted, Cd^+2^, Hg^+2^ complexes showed diamagnetic behaviors and are likely to possess an octahedral structure^31,50^.

Binuclear Co^+2^ complex (**3)** displayed two high intensity absorption bands at 600 and 510 nm, assignable to the ^4^T_1g_(F) → ^4^A_2g_(F)(ν_2_), ^4^T_1g_(F) → ^4^T_2g_(p) (*ν*_*3*_) transition, respectively, suggesting distorted octahedral geometry^51,52^. The μ_eff_ value for Co^+2^ complex = 3.28/ Co^+2^ B.M., that is lower than the spin-only value (3.87 B.M.,) assuring antiferromagnetic interaction^27,53^.

### Influence of γ-ray dose on electronic spectroscopy of chelates

The UV/Vis. spectra of γ-irradiated Cd^+2^ samples (**1R** and 1R*) at doses of 150, 200 kGy, respectively, exhibited nearly the same peaks for MLCT and π → π* at 510 and 224–220 nm as before gamma irradiation, while the n → π* transitions suffered blue shift and observed at 318 nm.

For γ-irradiated Hg^+2^ samples (**2R**), (2R*) at dose values of 150, 200 kGy, respectively, the UV/Vis. spectra displayed the same band of MLCT at 510 nm as before gamma irradiation, while the n → π* and π → π* transitions suffered red shift and observed at 334,320 and 220 nm, respectively. The findings indicated no geometry change under the influence of γ-rays.

### Thermal degradation

The thermograms for the complexes were performed from ambient until 800 °C with 10 °C/min heating rate in N_2_ atmosphere. The thermal stability was examined by thermogravimetric analysis (TG/DTG) and the data are given in Table 1 and depicted in Fig. 2.

**Table 1:** TGA findings of the metal complexes.

No	Compound	Temp. range/°C		Mass loss %		Reaction
		DTG	TG	Calc	F	
1	[CdL(OH)_2_(H_2_O)_2_]	–	30–250	–	–	- Steady part
		325, 481	250–717	85.83	85.54	-Decomp
		655	at 717	14.17	14.46^a^	≡ Cd
1R	[CdL(OH)_2_(H_2_O)_2_]	–	30–261	–	–	- Steady part
		322, 489,	261–799	85.83	85.65	-Decomp
		699	at 799	14.17	14.35^a^	≡Cd
1R*	[CdL(OH)_2_(H_2_O)_2_]	–	30–240	–	–	- Steady part
		320, 493	240–799	85.83	85.54	-Decomp
		636,702	at 799	14.17	14.46^a^	≡Cd
2	[HgLCl(H_2_O)_3_]Cl	–	30–250	–	–	Steady part
		254,267,301,422	250–555	100	100	-Decomp
			at 555	–	–	≡No residue
2R	[HgLCl(H_2_O)_3_]Cl	–	30–200	–	–	Steady part
		264,303,399,578	200–799	100	100	-Decomp
			at 799	–	–	≡No residue
2R*	[HgLCl(H_2_O)_3_]Cl	–	30–200	–	–	Steady part
		256,307,542	200–799	100	100	-Decomp
			at 799	–	–	≡No residue
3	[Co_2_L(NO_3_)_4_(H_2_O)_4_].4H_2_O	49	30–164	6.45	6.37	-4H_2_O
		184	164–220	10.57	10.78	-(4H_2_O + NO_2_)
		355,486	225–556	72.46	72.30	-Completation of Decomp
			at 556	10.52	10.55^a^	≡ 2Co

^a^Final product percent.

The TGA curves of both [CdL(OH)_2_(H_2_O)_2_](**1**) and [HgLCl(H_2_O)_3_]Cl **(2)** exhibited thermal stability till 250 °C. So, the existence of any solvent/water molecules may be ruled out. The analytical and spectroscopic data of these complexes is consistent with this observation. Decomposition of the complexes occurred within 250–717 and 250–550 °C for complexes (**1,2)**, respectively, yielding Cd metal as final product for complex (**1**) and no residue was obtained for complex (**2**).

The TGA curve of binuclear [Co_2_L(NO_3_)_4_(H_2_O)_4_].4H_2_O (**3),** indicated that the dehydration occurred within 30–164 °C range and involved the loss of four hydrated H_2_O molecules (calculated 6.45%, experimental 6.37%). The degradation started at 164 °C through loss of (4H_2_O + NO_2_) (calculated 10.57%, experimental 10.78%). The last part of complex gradually decomposed within 225–556 °C range (calculated 72.46%, experimental 72.30% weight loss). The complex completely decomposed at 556 °C and 2Co metal were left as residue (calculated 10.52%, experimental 10.55% weight loss). It is noted that [Co_2_L(NO_3_)_4_(H_2_O)_4_].4H_2_O complex (**3**) displayed the lowest thermal stability (D.T = 164°C), probably due to the oxidative effect of counter anion during decomposition steps together with the crowded structure of the binuclear complex.

### Thermal behavior of irradiated samples

The thermograms of the γ-irradiated Cd^+2^ samples (1R,1R*) (Fig. 2) displayed thermal stability until 261, 240 °C, respectively. The degradation process for both samples started at 261 and 240 °C and ended at higher temperature (799 °C) than for their parent sample (717 °C), remaining Cd metal as final residue in both cases. In DTG curves of (1R, 1R*), the endothermic DTG peaks were observed at 322, 489, 699 °C and 320,493,702 °C, respectively, while as, for parent complex (**1)** these peaks are observed at 325,480,654 °C. The findings indicated that 150 kGy irradiation dose may induced cross-linking leading to increase the thermal stability for sample (**1R**), whereas, 200 kGy irradiation dose may cause defect formation and decrease the thermal stability for sample (1R*).

The TG curves of γ-irradiated Hg^+2^ samples (**2R**,2R*) (Fig. 2**)** showed lower thermal stability than parent complex (**2)**. For both irradiated samples (**2R**,2R*), the decomposition process started at 200 °C and finished at 799 °C, while for parent complex (**2)** it started at 250 °C and completed at 555 °C, with no residue in all cases. The DTG curves of samples (**2R**,2R*) recorded endothermic peaks at 264,303,399,578 and 256,307,542 °C, respectively, while for parent complex (**2)** these peaks are observed at 253,301, 421 °C. TG/DTG findings indicated variation in the TG/DTG decomposition patterns especially for samples (**2R**,2R*). Also, as mention above the applied irradiation doses may cause defect formation leading to decrease the stability of samples **(2R**,2R*).

### Powder PXRD analysis

**Fig. 3:**
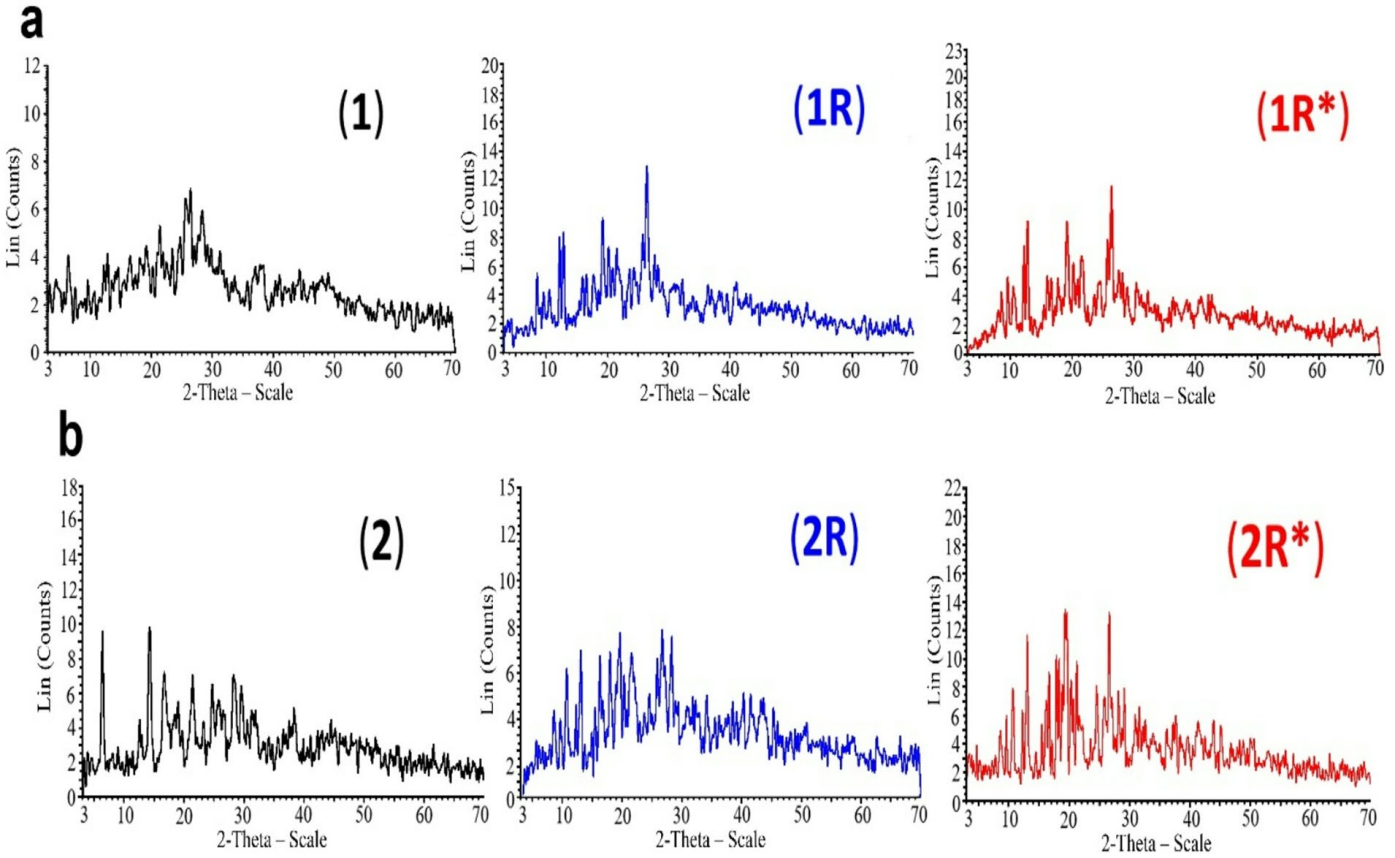
PXRD patterns of complexes a: (1,1R and 1R*) and b: (2,2R and 2R*).

To obtain more evidences for the exact structure of the prepared compounds and to explain the effect of γ-irradiation on their structures, X-ray diffraction was recorded for the Schiff base ligand and its Cd^+2^, Hg^+2^ complexes (**1,2**) as well as their γ-irradiated samples at 150 kGy (**1R, 2R**) and at 200 kGy (1R* and 2R*). The obtained X-ray diffractograms of the different crystalline powders are given in Fig. 3 and the important data are tabulated in Table 2.

**Table 2:** PXRD data for ligand, complexes (1, 1R, 1R*) and (2, 2R, 2R*).

Compound	2θ^o^	d-spacing (Å)	Rel. Intensity (%)	Average cry. size (nm)	dislocation density (${\varvec{\delta}}$) nm^-2^
**L**	19.2	4.607	100	43.7	0.000524
	24.512	3.628	97.3		
	18.291	4.846	53.1		
	12.772	6.925	48.4		
**1**	25.506	3.489	94.7	48.9	0.000418
	28.25	3.156	87.2		
	21.441	4.140	77.1		
	26.42	3.370	100		
**1R**	12.129	7.291	61.7	21.1	0.00225
	12.751	6.937	64.3		
	26.391	3.374	100		
	19.155	4.629	71.3		
1R*	12.675	6.978	79.3	28.9	0.001197
	19.112	4.640	78.6		
	26.322	3.383	100		
	12.092	7.313	64.2		
**2**	6.312	13.990	97.6	25.0	0.0016
	14.176	6.242	100		
	16.665	5.315	72.8		
	21.34	4.160	72.4		
**2R**	12.993	6.808	88	25.6	0.001526
	26.624	3.345	100		
	19.612	4.522	97.7		
	17.885	4.955	86.9		
2R*	12.974	6.818	87.7	17.1	0.003419
	19.563	4.533	99.3		
	26.608	3.347	99.7		
	19.256	4.605	100		

The PXRD pattern of the ligand exhibited intensity peaks centered at 2*θ* = 9.6°, 12.7°, 18.29°, 19.2° and 24.5° (Table 2, Fig. 3S). It is noted that the peak observed at *2θ* = 9.6° in the PXRD of the ligand is disappeared in the complexes (**1**,**2**) and instead a high intensity new peak is appeared at 2θ = 6.3^o.^, this peak disappeared in the irradiated samples (**1R**,1R*) and (**2R**,2R*). The PXRD of the irradiated samples (**1R**,1R*) (Fig. 3) showed the existence of number of new peaks at *2θ* = 8.4°, 9.5^o^ and 10.4° with significantly increasing in the peak intensities with increasing the irradiation dose, indicating a movement towards higher crystallinity upon irradiation for samples (**1R**,1R*).

The observed high intensities’ new peaks with maxima at *2θ* = 8.5°, 9.5°, 10.6°, 16.3°,17.8° and 26.6 ^o^, in the PXRD of the irradiated samples (**2R**, 2R*) confirming their higher degree of crystallinity. It is worth to mention that, the peak intensity of sample **2R** is greater than 2R*. The crystallite size of the compounds, *D*_*XRD*_, was computed from PXRD diffractograms via Scherer’s formula^54,55^. *D*_*XRD*_ = *(0.95λ)/β (cos θ),* where *D*_*XRD*_ is the mean crystallite size, *λ* is the wavelength, *β* is full-width of half maximum, *θ* is angle of diffraction peak. The calculated particle size for ligand, Cd^+2^ and Hg^+2^ complexes (**1**,**2**) equals 43.7, 48.9 and 25.0 nm, respectively. This suggests that the ligand and its complexes are nanocrystalline^56^. The particle size of the irradiated samples (**1R**,1R*) equals 21.1 and 28.9 nm and for (**2R**, 2R*) samples is 25.6 and 17.1 nm, respectively. The particle size was altered upon irradiation at doses 150 and 200 kGy. The dislocation density *δ* value of complexes is calculated from the value of mean crystallite size *D* by the formula $\delta =1/{D}^{2}$ ^57^. The computed value of *δ* for complexes (**1**,**2**) is 0.000418, 0.0016 nm^−2^, respectively, and 0.0104 and 0.00312 nm^−2^ for the irradiated (**1R**,1R*) samples. The computed value of *δ* for (**2R**, 2R*) samples is 0.001526 and 0.003419 nm^−2^, respectively. The stress factor that irradiation induces is responsible for the observed alter of the crystallite size, the intensity of peaks and dislocation density.

**Fig. 4:**
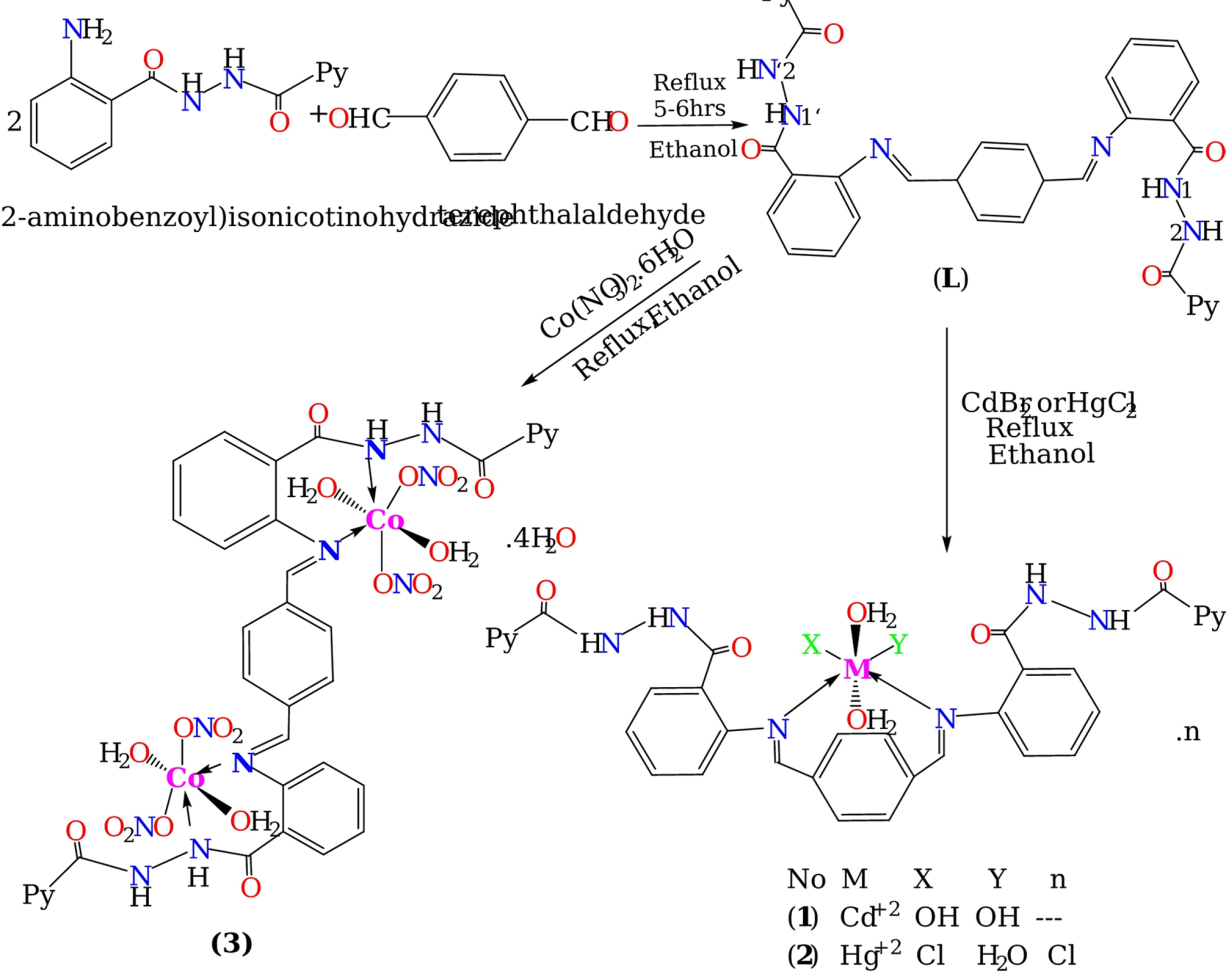
Synthesis of ligand (**L**) and its metal complexes (**1,2,3**).

By using the aforementioned data, the proposed structure of the compounds was drawn in Fig. 4.

### Biological assessments

#### Cytotoxicity evaluation

**Fig. 5:**
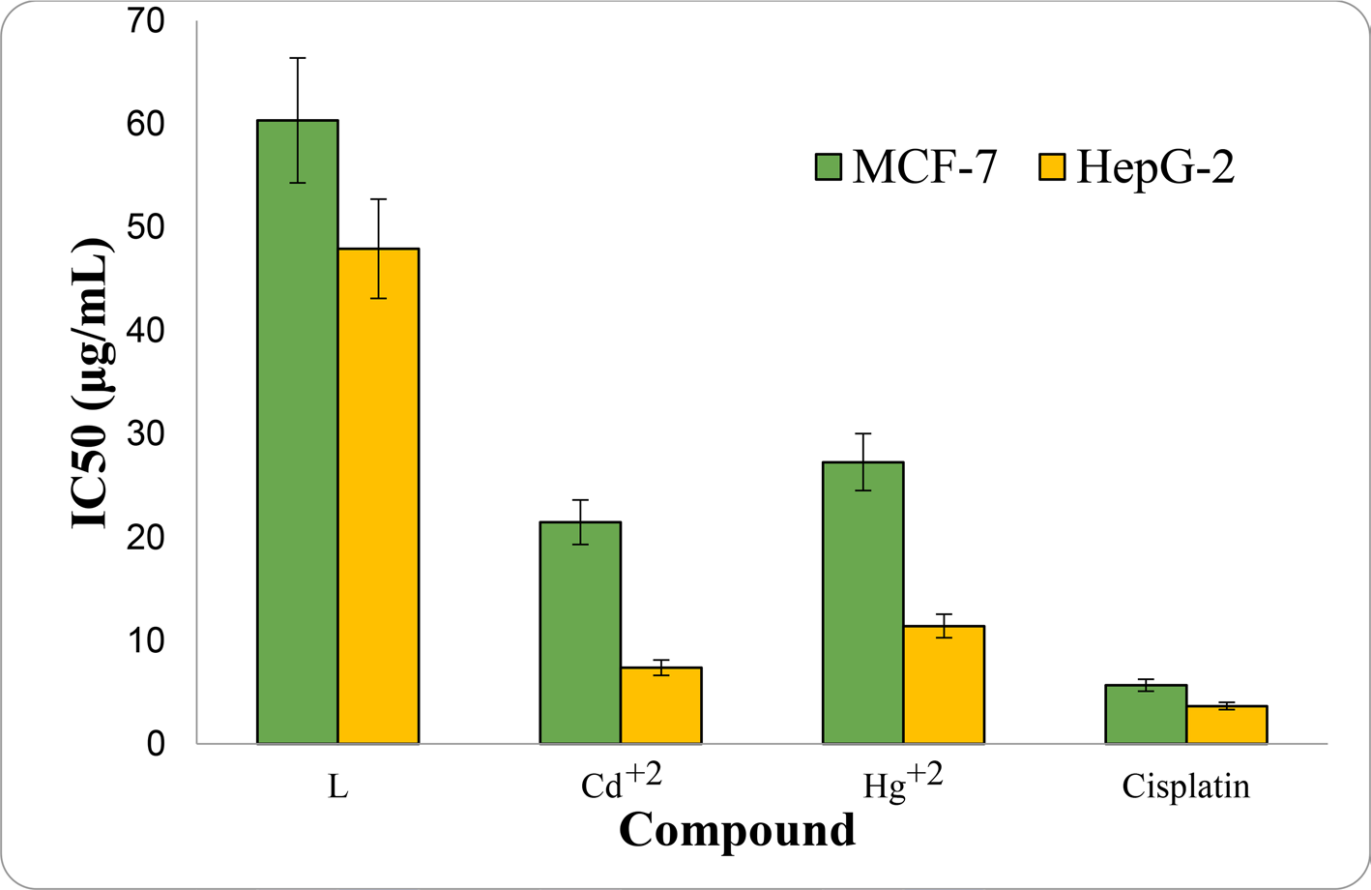
IC_50_ representation of ligand (**L**) and its Cd^+2^ and Hg^+2^ complexes against MCF-7 and HepG2.

The cytotoxicity of the Schiff base ligand and its parent Cd^+2^, Hg^+2^ chelates (**1**,**2)** was evaluated compared to Cisplatin as a standard drug positive control. In vitro screening was tested towards MCF-7 and HepG-2 cancer cell-lines. The computed IC_50_ values of the evaluated compounds are tabulated in Table 3 and drawn in Fig. 5. All values are shown as mean ± standard deviation of three independent experiment. From this screening assay, it came out that the examined metal chelates possess better activity than the present ligand; this indicates the improvement of anticancer activity after coordination^58^. Cd^+2^ complex (**1**) exhibited the supreme cytotoxic activity towards both cell lines MCF-7 and HepG-2 with IC_50_ = 21.49 ± 2.13, 7.38 ± 0.64 µg/mL, respectively, followed by Hg^+2^ complex (**2**) IC_50_ = 27.28 ± 2.06, 11.42 ± 1.04 µg/mL, respectively then, the ligand; IC_50_ = 60.35 ± 4.11, 47.91 ± 2.39 µg/mL, respectively compared to cisplatin (IC_50_ = 5.69 ± 0.47, 3.68 ± 0.18 µg/mL). The general order of the activity for both cell lines is: Cisplatin > Cd^+2^ (**1**) > Hg^+2^ (**2**) > L.

**Table 3:** IC_50_ of ligand and its complexes on MCF-7 and HepG-2 cell lines with cisplatin as a standard reference.

No	Compound		IC_50_(μg/mL)a	References
		MCF-7	HepG-2	
–	L	60.35 ± 4.11	47.91 ± 2.39	Our work
1	[CdL(OH)2(H_2_O)_2_]	21.49 ± 2.13	7.38 ± 0.64	Our work
2	[HgLCl(H_2_O)_3_]Cl	27.28 ± 2.06	11.42 ± 1.04	Our work
–	Cisplatin	5.69 ± 0.47	3.68 ± 0.18	Our work
	[Cd(BCA)(PHN)(H_2_O)]2Cl⋅2H_2_O	29.97	–	^57^
	[Cd(ANA)_2_Cl_2_]	40.88 ± 0.34	–	^58^
	[Hg(ANA)_2_Cl_2_]	33.29 ± 0.12	–	^58^
	PTIH ([Hg(SCN)_2_(HL)]	–	24	^59^

^a^IC_50_ value is the concentration at which 50% survival of cells was observed.

The finding acertain that the tested complexes displayed improved cytotoxic activities against the two tested cell lines relative to the free ligand. The observed anticancer activity of the tested compounds agrees well with the documented activity of some other work reported earlier^58–60^. the different metal ion, particle size of the metal ion, the difference in the co-ligand in the coordination sphere, the counter anion, leads to their difference in antiproliferative activity^59^. The additional important factor is the particle size used for the activity is in the nanometer scale which enhances the anticancer property through easy permeation of compounds into cancer cells^61^. However, in vivo investigations are necessary to understand the mechanism of the anti-proliferative activity of these complexes.

#### Antimicrobial activity

##### Antibacterial activity

**Fig. 6:**
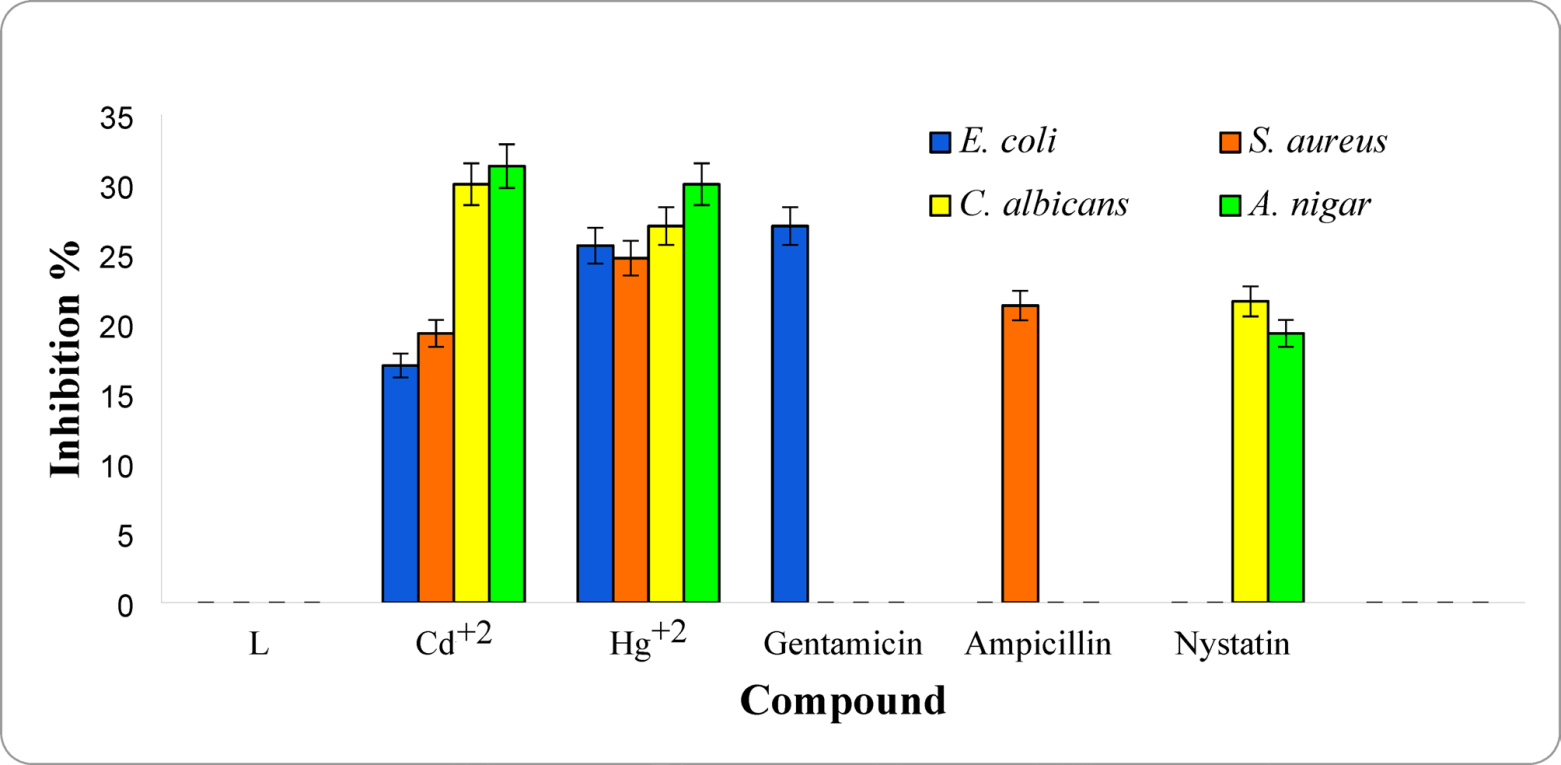
Antimicrobial property for ligand (**L**) and its Cd^+2^ and Hg^+2^ complexes.

The Schiff base ligand and its Cd^+2^, Hg^+2^ chelates (**1**,**2**) were studied for their antibacterial activity against *E. coli* and *S. aureus*. For comparison purposes, Gentamicin and Ampicillin as standard drugs were also tested for their antibacterial property. The outcomes are grouped in Table 4 and shown in Fig. 6. The present ligand showed no activity against the tested microorganisms, whereas Cd^+2^, Hg^+2^ chelates (**1**,**2**) showed significant activity against both tested bacterial strains on comparing with the standard drug. For bacterial strain *E. coli*, Hg^+2^ complex (**2**), showed excellent activity (25.6 mm), while Cd^+2^ complex, showed moderate activity (17 mm) compared with standard bactericide Gentamicin (27 mm). For *S. aureus* strain*,* Hg^+2^ complex showed excellent antibacterial activity (24.7 mm) greater than the standard bactericide ampicillin (21.3 mm) also, Cd^+2^ complex (**1**) showed significant activity (19.3 mm). The antibacterial property towards *E. coli* and *S. aureus* can be ordered as: Gentamicin > Hg^+2^ (**2**) > Cd^+2^ (**1**) For *E. coli*

$${\text{Hg}}^{{ + {2}}} \left( {\mathbf{2}} \right) \, > {\text{ Ampicillin }} > {\text{ Cd}}^{{ + {2}}} \left( {\mathbf{1}} \right){\text{ For}}S. \, aureus$$

**Table 4:** Antimicrobial activity of ligand and its metal complexes (**1,2**).

No	Compound	Zone of inhibition (mm)				*P*-Value	ReferenceS
		Bacteria		Fungi			
		*E.coli* *(ATCC:10,536)*	*S. aureus* *(ATCC:13,565)*	*C.albicans* *(ATCC:10,231)*	*A.nigar* *(ATCC:16,404)*		
	Gentamicin	27.0 ± 1.0^c^	–	–	–	< 0.001	Our work
	Ampicillin	–	21.3 ± 0.6^b^	–		< 0.001	Our work
	Nystatin	–	–	21.6 ± 0.6^a^	19.3 ± 0.6^a^	< 0.001	Our work
	**L**	NA	NA	NA	NA	< 0.001	Our work
**1**	[CdL(OH)_2_(H_2_O)_2_]	17.0 ± 1.0^a^	19.3 ± 0.6^a^	30.0 ± 1.0^c^	31.3 ± 0.6^c^	< 0.001	Our work
**2**	[HgLCl(H_2_O)_3_]Cl	25.6 ± 0.6^b^	24.7 ± 0.6^c^	27.0 ± 1.0^b^	30.0 ± 1.0^b^	< 0.001	Our work
	Cd(II) complex	13	14	NA	n.d		63
	[Cd(C_9_H_10_N_2_)_2_Cl_2_]	16	n.d	n.d	n.d		67
	[Hg(H_2_L)(H_2_O)Cl]	18	15	14	n.d		66
	[Hg(C_9_H_10_N_2_)_2_Cl_2_]	16	n.d	18	10		67
	[Hg_2_(HL)Cl_4_].EtOH	–	–	16	21		38

Zone of inhibition is expressed in the form of Mean ± Standard deviation (mm).—Well diameter (6 mm).

NA: No activity.—100µL was tested.

Values that share the same letter at the same row are not significant.

Values that share different letters at the same row are significant.

n.d.: Not determined.

##### Antifungal activity

The ligand and its Cd^+2^, Hg^+2^ complexes (**1**,**2**) were also studied for their antifungal activity towards *C. albicans* and *A. nigar,* with Nystatin as reference drugs (Fig. 6)*.* The finding indicated that the ligand possesses no activity against the two tested fungal strains. Cd^+2^, Hg^+2^ complexes (**1**,**2**) showed excellent antifungal activity (30 and 27 mm) against *C. albicans* and (31.3 and 30 mm) against *A. nigar,* respectively*,* greater than the standard antifungal drug Nystatin (21.6, 19.3 mm).

The antifungal property can be ordered as: Cd^+2^ (**1**) > Hg^+2^ (**2**) > Nystatin, for both *C. albicans and A. nigar.* Such high in bio-activity of the Cd^+2^, Hg^+2^ complexes (**1**,**2**) can be demonstrated by Overtone’s principle and Tweedy’s chelation concept^52^. Accordingly, the lipophilicity of metal ion enhanced and hence facilitates diffusion of complexes into the bacterial cells making it easy to kill many more microorganisms. The finding showed that the [HgLCl(H_2_O)_3_]Cl (**2**) showed significant antibacterial activity against the tested microorganisms. The finding also showed that both [HgLCl(H_2_O)_3_]Cl and [CdL(OH)_2_(H_2_O)_2_] had excellent antifungal activities greater than the standard drugs against the tested microorganisms. The observed antimicrobial activity of the obtained compounds agrees with the documented activity of some other work reported earlier^62–66^. Therefore, these complexes can be employed as more effective drug research attention in the near future for pathogenic microbial diseases^62–64^.

## Conclusions

In the current work, new bis-Schiff base ligand (**L**) and its mononuclear Cd^+2^, Hg^+2^ (**1**,**2**) and binuclear Co^+2^ (**3**) chelates have been fabricated and established by various analytical, spectroscopic and physical techniques. The influence of γ-irradiation at dose values of 150 and 200 kGy and at 2.2 kGy h^−1^ dose rate, on their physical and structural properties was discussed. All chelates possess octahedral stereochemistry. No alternation in their stereo structure was detected post irradiation. The TG thermographs showed that the thermal stability of Cd^+2^ sample (**1R**) was enhanced post *γ*-irradiation and the binuclear Co^+2^ complex (**3**) showed the lowest thermal stability. The in vitro anticancer property revealed that the tested complexes possessed activity better than the free ligand and their activity towards HepG-2 is better than MCF-7 cell lines. [CdL(OH)_2_(H_2_O)_2_] was the most active one towards both cells. The ligand and its Cd^+2^, Hg^+2^ chelates (**1**,**2**) had been also screened against some bacterial and fungal species. The finding revealed that the [HgLCl(H_2_O)_3_]Cl (**2**) showed excellent antibacterial activity and [CdL(OH)_2_(H_2_O)_2_] **(1**) showed excellent antifungal activity, greater than the standard drugs against the tested microorganisms.

## Electronic supplementary material

Below is the link to the electronic supplementary material.

Supplementary Material

## Data Availability

Data is provided within the manuscript or supplementary information.
