# Supplementary material for: Fabrication, spectroscopic inspection, biological evaluation of some transition metal complexes with bis- azomethine ligand and γ-ray irradiation dose effect
Source: Sci Rep. 2025 Jul 16;15:25670. doi: 10.1038/s41598-025-08660-5 (PMC12263966; doi:10.1038/s41598-025-08660-5)
Supplement: Supplementary file 1 — Supplementary Material [file 41598_2025_8660_MOESM1_ESM.docx]

**Supplementary** **informations**

**Fabrication, spectroscopic inspection, biological evaluation of some transition metal complexes with *bis*- azomethine ligand and γ-ray irradiation dose effect**

Hanaa A. El-Boraey^1*^ Ohyla A. EL-Gammal^2^

[^1^Department of Chemistry, Faculty of Science, Menoufia University, Shebin El- Kom, 32511, Egypt.^2^Department of Pathology, University Hospital, Menoufia University, Shebin El- Kom,32511, Egypt. E-mail address:[hanaaelborai@science.menofia.edu.eg](mailto:hanaaelborai@science.menofia.edu.eg) (Hanaa A. El-Boraey)].

***1.Instrumentations***

The microanalyses (C, H, N) were achieved at Cairo University, Micro Analytical Center, using CHNS-932 (LECO) Vario Elemental Analyzer. Metal and halide ions of metal complexes were determined using the standard methods. The infrared spectra of ligand and its complexes were obtained using Nenexeus-Nicolidite-640-MSA FT-IR spectrophotometer (4000-400 cm^-1^), Thermo-Electronics Co in KBr discs. The ^1^H NMR spectrum was recorded in DMSO-*d_6_* solvent at room temperature using Varian Gemini 200 NMR spectrophotometer at 300 MHz. In addition, the UV-Visible absorption spectra were measured in ethanol solvent using 4802 UV/Vis double beam spectrophotometer. The molar room temperature conductivity of studied complexes was estimated in DMF (10^-3^ M) using a CON 6000 conductivity meter. At room temperature, magnetic susceptibilities of studied complexes were measured by the modified Gouy method using magnetic susceptibility Johnson Matthey balance. The effective magnetic moments were calculated using the formula μ_eff_ = 2.828(χ_m_T)^1/2^ B.M., where χ_m_ is the molar susceptibility corrected for diamagnetism of all atoms in the compounds. Thermal analyses (TGA/DTG) were carried out by using a Shimadzu DTG/TG-50 thermal Analyzer with heating rate 10°C/ min in nitrogen atmosphere with a flowing rate 20 ml/min in the temperature range 28-800°C using platinum crucibles. X-ray diffraction (XRD) patterns for complexes **(1,2)** and γ-irradiated (**1R, 2R**), (**1R***, **2R***) of solid powder samples were carried out by BRUKER Co. D8 ADVANCE at the central metallurgical research and development institute (CDRDI), Eltbeen, Helwan, Cairo, Egypt.

***2.Biological studies***

***2.1. In vitro*** ***cytotoxicity evaluation***

The breast carcinoma cell line (MCF-7) and hepatocellular carcinoma (HepG-2) cell lines were obtained from the American Type Culture Collection (ATCC, Rockville, MD) and investigated at the Regional Center for Mycology and Biotechnology, Al-Azhar University, Cairo, Egypt.

For antitumor assays, the tumor cell lines were suspended in medium at concentration 5x10^4^ cell/well in Corning 96-well tissue culture plates, then incubated for 24 h. The tested compounds were then added into 96-well plates (three replicates) to achieve twelve concentrations for each compound. Six vehicle controls with media or 0.5 % DMSO were run for each 96 well plate as a control. After incubating for 24 h, the numbers of viable cells were determined by the MTT test.

Briefly, the media was removed from the 96 well plate and replaced with 100 µL of fresh culture RPMI 1640 medium without phenol red then 10 µL of the 12 mM MTT stock solution (5 mg of MTT in 1 mL of PBS) to each well including the untreated controls. The 96 well plates were then incubated at 37 °C and 5% CO_2_ for 4 hrs. An 85 µL aliquot of the media was removed from the wells, and 50 µL of DMSO was added to each well and mixed thoroughly with the pipette and incubated at 37 °C for 10 min. Then, the optical density was measured at 590 nm with the microplate reader (SunRise, TECAN, Inc, USA) to determine the number of viable cells and the percentage of viability was calculated as [(ODt/ODc)]x100% where ODt is the mean optical density of wells treated with the tested sample and ODc is the mean optical density of untreated cells. The relation between surviving cells and drug concentration is plotted to get the survival curve of each tumor cell line after treatment with the specified compound. The 50% inhibitory concentration (IC_50_), the concentration required to cause toxic effects in 50% of intact cells, was estimated from graphic plots of the dose response curve for each conc. using Graphpad Prism software (San Diego, CA. USA).

***2.2. Antimicrobial assay***

***2.2.1.Method of testing***

The sterilized media was poured onto the sterilized Petri dishes (20-25) mL, each petri dish) and allowed to solidify at room temperature. Microbial suspension was prepared in sterilized saline equivalent to McFarland 0.5 standard solution (1.5x 10^5^ CFU mL^-1^) and its turbidity was adjusted to OD= 0.13 using spectrophotometer at 625 nm. Optimally, within 15 min. after adjusting the turbidity of the inoculum suspension, a sterile cotton swab was dipped into the adjusted suspension and was flooded on the dried agar surface then allowed to dry for 15 min. with lid in place. Wells of 6 mm diameter was made in the solidified media with the help of sterile borer. 100 μL of the solution of the tested compound was added to each well with the help of micropipette. The plates were incubated at 37 °C for 24 h in case of antibacterial activity. This experiment was carried out in triplicate and zones of inhibition were measured in mm scale.


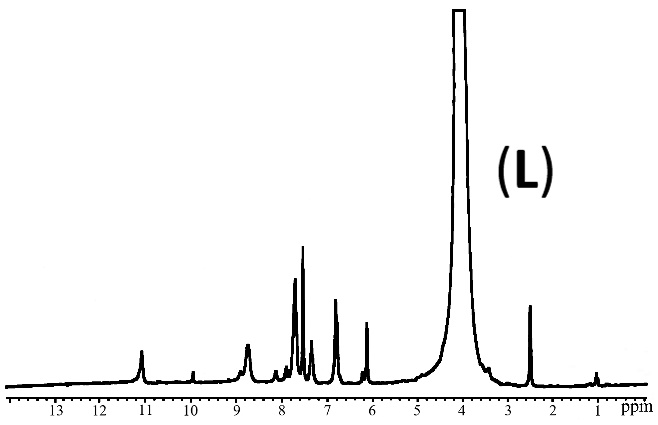


**Fig.1S:** ^1^H-NMR of ligand (L)


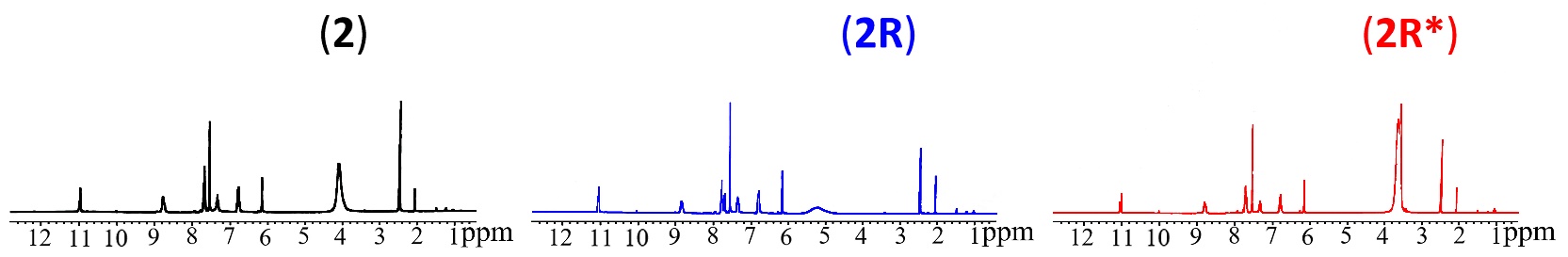


**Fig.2S: ^1^**H-NMR of complexes **(2,2R** and **2R*).**


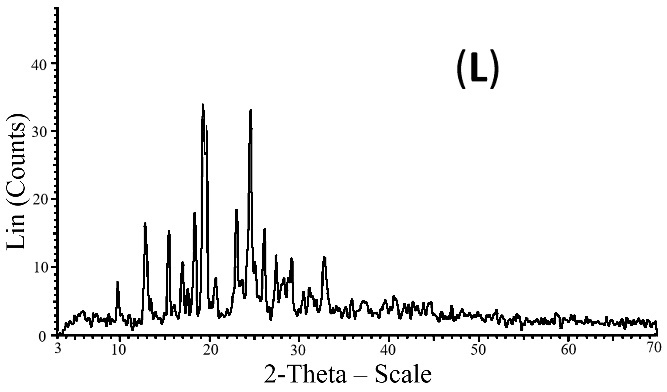


**Fig.3S:** PXRD patterns of ligand (**L**)
